# The Medical Ward Sister: "A Sermon on the Mount."

**Published:** 1917-12-22

**Authors:** 


### SOME HOSPITAL TYPES OF BRITISH NURSING.

### III. The Medical Ward Sister: "A Sermon on the Mount."

Looking back upon the impressions of my earlier hospital days, none stand out so clearly as those made by the ward sisters under whom I worked. Amongst many of varying types one stands supreme. A small dainty woman, with dark hair and searching eyes—for all her smallness, great dignity of manner; quiet tread, though never so quiet as not to be heard; gentle voice, often very tender, but able at need to speak with authority.

This sister ruled a large and very heavy ward, where great pressure often arose. It was in the days when medical wards were often heaviest, especially in the winter, when many typhoid cases were nursed in general wards.

Yet no matter how much work there was to be done, nor how often at the end of a busy "take-in" Sister might be asked to put up extra beds (allowed in those far-off days without restriction), there was never a sigh of wearinesss nor a murmur of discontent.

To Sister the patient never arrived at an inconvenient moment, she gave the impression that she was waiting, so pleasant was her welcome to the patient, so courteous her manner to the friends, however worrying or tiresome they might be.

Whatever could be done for the patients was so willingly performed, and Sister, by her influence, led all her nurses to think first of those under her care.

Was there any very difficult or distasteful task, Sister would take an active part in doing it—she never allowed her exalted position to serve as an excuse for putting aside what was uncongenial, and her way of overcoming difficulties made those working under her alert to ignore their own small trials.

The work always went on smoothly. To Sister the hospital ward was the home, her patients her honoured guests, and her staff her willing helpers.

Sister could always draw out the best in everyone. Her care of her nurses and her interest in their training was as great as her kindness to her patients.

The new probationer, the inexperienced "pro-staff," was never made to feel that she was a burden to be patiently borne with.

Every possible opportunity of teaching, or of giving a cheering word of encouragement, was taken advantage of. Were it necessary to reprimand, it might be sternly, even severely given, but always unheard by others, and then the better way pointed out so gently, with a smile of faith as it were for the future, that the uphill path to success seemed still to be possible.

Sister never talked religion, but she had so grasped Florence Nightingale's ideal that a nurse should *be* a "Sermon on the Mount" that her very presence was inspiring.

She worked for many years, doing noble work for the poor in the East End, and numbers blessed her, but far beyond the ken of one so humble-minded, the influence of her life in the hospital spread out, and junior workers leaving her ward took high ideals with them, and a determination to persevere in realising them.

Londoner.

